# Team Cognizant Action: Insights into Developing Team Synergies in Sports

**DOI:** 10.1186/s40798-026-01045-6

**Published:** 2026-07-09

**Authors:** Daniel Carrilho, Henrique Lopes, Duarte Araújo

**Affiliations:** CIPER, Faculdade de Motricidade Humana, Universidade de Lisboa, Estrada da Costa, Cruz Quebrada-Dafundo, 1495-688 Lisbon, Portugal

**Keywords:** Team sports, Team behavior, Shared affordances, Coordinated effectivities, Team cognition

## Abstract

Team cognition and collective intelligence describe how individuals in a team interact to achieve shared goals. In team sports, these concepts are crucial for understanding how players coordinate their actions in dynamic match environments. Inferential psychological approaches treat team cognition as an internal mental process and coordinated action as its execution, with limited explanation of how players continuously regulate actions to sustain collective goal-directed behavior. To address this, we propose a reconceptualization of team behavior grounded in the *ecological dynamics* framework called *team cognizant action*. We argue that coordinated action is team cognition expressed through direct perception and action couplings between teammates and the match environment. By shifting the unit of analysis to the team–environment system, defined as the interdependent relationship between players and their performance environment within a task, we offer a parsimonious and comprehensive account of team behavior. Within this system, coordination emerges from how players detect ecological information specifying opportunities for collective action. To act on such opportunities requires coordination among players’ skills to achieve shared goals. Such coordinated behavior underlies *team synergies* which in turn operationalize team cognizant action. Consequently, to foster cognizant action, practice sessions should emphasize representative learning design to enhance shared perceptual attunement and collective movement calibration.

**Supplementary Information:**

The online version contains supplementary material available at 10.1186/s40798-026-01045-6.

## Key Points

- Team cognizant action describes how team cognition and action are indistinguishable, rather than separated.
- Team cognizant action is guided by opportunities for collective action offered by the performance environment.
- Team synergies are an expression of team cognizant action, emerging as players coordinate with each other during sports matches toward shared goals.
- Enhancing cognizant action implies designing training tasks that correspond to match functions.

**Supplementary Information:**

The online version contains supplementary material available at 10.1186/s40798-026-01045-6.

## Introduction

Collective intelligence and team cognition are commonly used terms to define how interdependent individuals cooperate to achieve shared goals [1, 2]. Both concepts are typically associated with overlapping knowledge structures formed across individuals within a group that predict their behavior during a task [3]. Because of limitations in explaining how these structures would be stored, updated, and shared across individuals, traditional approaches have been criticized as insufficient to fully account for team coordination in dynamic environments [4–6]: *“*…this traditional conceptualization of team cognition, with an emphasis on knowledge that is relatively static or unchanging, does not account for the more dynamic cognition of a team in a constantly changing environment*"* [4, p. 415].

Consequently, more recent approaches propose that the ‘ontogenesis’ of a team is predicated on shared goals, which differentiates teams from groups [7]; that team cognition is an activity, not a property or product, and is inseparable from context [4]; and that team behavior is best studied when considering the team as the unit of analysis [5], focusing on communication channels and interaction processes [2].

This move toward a more situated and goal-directed analysis of team behavior calls for a reconceptualization of how team cognition regulates coordinated group action, which is obscured by the dominant assumption that cognition (and intelligence) resides in mental mechanisms, while action is treated as its downstream execution. In this paper, we challenge this separation by adopting an ecological dynamics approach [8], which offers a parsimonious account of cognition as inseparable from action [9].

Ecological dynamics [8, 10], a theoretical framework grounded in ecological psychology [11], has been central to this reconceptualization, rejecting the separation between cognition and action and instead advancing a theory of *cognizant action*—action is not a mere motor execution following mental predictions but is itself imbued with cognition [10].

Team sports provide an ideal setting to confront this paradigmatic separation between cognition and action. In this paper, we use the example of football teams to advance a theory of cognizant action as it applies to team behavior: what we term *team cognizant action*. We propose that team cognizant action emerges from the direct perception of shared affordances, defined as opportunities for joint action specified by ecological information and available to multiple players [12]. This extends the ecological psychology concept of affordances, defined as opportunities for action offered to an organism by the environment [11], to the team level.

Shared affordances guide coordinated development of team effectivities, defined as the functional coupling of players’ action capabilities. Following Shaw [13], effectivities refer to the action capabilities of an organism that determine which affordances can be realized; here, this concept is extended to capture how players coordinate their capabilities with possibilities for action provided by the match (see also [14–16]).

In the following sections, we provide theoretical grounding and empirical evidence to support a conceptualization of team cognizant action within the dynamic and goal-directed reorganization of the team–environment system, defined as the interdependent relationship between players and their performance environment within a task. Our central argument is that team cognition is not a separate mental process that interacts with coordinated team action, but it is rather embodied and embedded in the coordination between players’ actions as they exploit the match environment through whole-body action [9].

This perspective shifts the unit of analysis from the team as an isolated entity to the team–environment system, within which team cognizant action is expressed through team synergic properties defined as measurable patterns of functional coordination that reflect how players maintain group behavior as a coherent unit [17–19]. Because team cognizant action is expressed within the team–environment system, we propose that team performance is situated and can be monitored by the continuous analysis of team synergic properties. Thus, in the final section we highlight how team expertise is developed through shared perceptual attunement (i.e., increased sensitivity to task-relevant informational variables) and coordinated team movement calibration (i.e., scaling actions to those informational variables) during practice tasks that are representative of the match [20–22].

## Symbolic and Ecological Information

Recent theoretical perspectives on team behavior share common ground, namely, that teams are formed by interdependent individuals who interact within a performance context in pursuit of shared goals [4, 7, 23]. However, these perspectives diverge in how they understand the nature of information and the processes involved in team–environment interactions [24, 25]. To clarify the ecological dynamics approach adopted in this paper, we briefly contrast it with inferential approaches to cognition, drawing on examples such as Team Mental Models [5] and Team Dynamics Theory [7].

Team dynamics theory proposes that team behavior can be explained through a nomological network linking cohesion (shared goals), team mental models, coordination, and collective efficacy [7]. Within this framework, team mental models are conceptualized as structured knowledge representations that must be aligned among teammates through communication, shared task engagement, and feedback to support effective coordination [5]. Such approaches have been particularly useful for explaining how shared knowledge structures contribute to role clarity, communication, and strategic alignment in team settings.

Inferential approaches are generally grounded in the assumption that information from the environment is ambiguous (i.e., impoverished), requiring internal processing to generate meaningful percepts [26]. From this perspective, sensory stimulation is transformed into internal representations through cognitive mechanisms, which organize and interpret environmental input to support action (i.e., information-processing in the mind) [27]. In contrast, approaches grounded in ecological realism [11, 28], such as ecological dynamics, propose that information exists in the ecology and it is inherently meaningful for an active organism, as it is specified in ambient energy patterns (e.g., light) that can be directly perceived without the need for intermediate inferential processes. This distinction between symbolic [29] and ecological [11, 28] information reflects a broader theoretical difference regarding whether perception is indirect (mediated by internal representations) [30] or direct [31].

Symbolic information, as formalized within Shannon’s [29] framework, is defined in terms of statistical structure and uncertainty reduction, and is, therefore, non-semantic (i.e., does not carry meaning about what it refers to). It is abstracted from the rate-dependent dynamics of the environment and does not preserve a lawful relation to the physical processes generating it. In contrast, from an ecological perspective, environmental properties are directly specified in detectable patterns of ambient energy, what is termed ecological information [11].

Ecological information is specified in the spatiotemporal structure of energy arrays and remains directly coupled to environmental properties, enabling perception to be direct and action-relevant. The ambient energy array, formed by lawfully structured information (e.g., electromagnetic waves that form light patterns, which differ across objects, that can be detected by the human visual system ), directly reveals the environment (e.g., players, ball, pitch surface, goalposts) for players who actively perceive through movement , such as moving toward the goal, moving to create space for the ball carrier , or avoiding a defender approaching.

From an ecological dynamics perspective, this distinction has important implications for how team coordination is explained. Rather than relying on internally constructed representations, coordinated team behavior is understood to emerge from the continuous coupling between performers and their environment through directly perceived information. In this sense, inferential and ecological dynamics approaches are ontologically distinct, because they rely on different assumptions about the nature of information and the relation between perception and action.

One key example of this lawfully specified information is *optic flow*: the structured pattern of light changes across the retina as an observer moves through the environment. Optic flow is not merely visual motion but a richly informative array that specifies layout and affordances, enabling players to act accordingly and perceive their own movement and the movement of their teammates relative to the environment. This enables the formation of team synergies through the perception of  shared affordances and realizing them through coordinated effectivities [32, 33].

## Shared Affordances and Coordinated Effectivities

To play together in the match environment, players perceive and act on shared affordances—opportunities for group action [12]—to achieve team goals. Shared affordances are perceived when the same ecological information guides multiple players’ actions relative to their effectivities (i.e., perceptual-motor skills) [17].

**Fig. 1:**
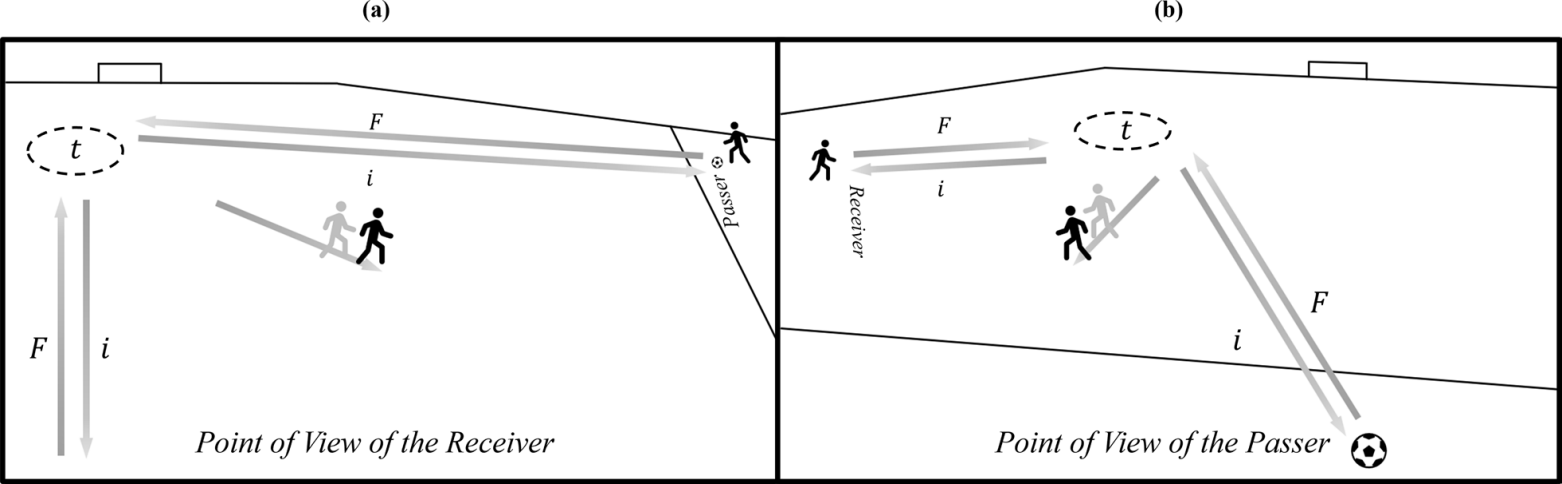
Coordinated passer–receiver actions for a shared affordance. An attacker (black) dragging a defender (gray) reveals a space that is perceived by teammates (passer and receiver), at different points of observation, as a shared affordance to move the ball to a target location ($t$). From each point of view, the receiver’s (**a**) and the passer’s (**b**), both players perceive the same ecological information ($i$) specifying how they should interpersonally coordinate their actions ($F$)

Figure 1 illustrates an attacking situation, showing how attackers in different locations on the pitch can perceive a shared affordance by detecting that both they and their teammates can act on the same ecological information.

In this situation, an attacker dragging a defender (gray) generates changes in the environment (variants in optic flow), revealing a target space ($t$) between defenders. This ecological information ($i$), such as distance and orientation to the target space, specifies a shared affordance for ball progression. To act upon this shared affordance, attackers must coordinate their actions: the ball carrier perceives the opportunity to pass, while a teammate perceives the opportunity to exploit space. As both act on this shared affordance, their movements become coordinated (coordinated effectivities), resulting in a synchronized pass-and-run action (emerging synergy) that enables progression toward the goal (performance outcome) [32].

Both the passer (Fig. 1a) and the receiver (Fig. 1b) detect this ecological information from their respective viewpoints and act upon it by coordinating their effectivities: the passer calibrates the force (*F*) applied to the ball based on the location of the target space and the receiver’s movement, while the receiver continuously adjusts their movement, producing force (*F*) against the ground by accelerating, decelerating, or maintaining speed, to intercept the ball. Through this shared perceptual attunement to the same information source and to each other’s affordances, their actions become functionally coupled, forming a coherent unit that realizes the shared affordance.

This example demonstrates that players can perceive shared affordances, because they can perceive affordances for their teammates, which entails perceiving their action capabilities (effectivities). This claim is supported by empirical research showing that individuals can accurately perceive others’ abilities (e.g., jumping ability) from movement patterns, such as gait [34]. With practice, this ability is further developed as players become attuned to teammates’ effectivities, enabling more effective coordination [12]. Despite limited empirical work directly linking shared affordances and coordinated effectivities in collective performance environments, Carrilho et al. [35] showed that football players regulate movement kinematics (e.g., speed, direction changes) according to specific affordances, supporting the role of ecological information in regulating action.

In summary, shared affordances refer to opportunities for coordinated action specified by ecological information and available to multiple players sharing common goals, while coordinated effectivities refer to the functional coupling of those players’ action capabilities [36, 37]. The perception of  shared affordances and their realization through coordinated effectivities allows the formation of team synergies that express team cognizant action.

## Team Cognizant Action Expressed by Team Synergies

Direct shared perception is coupled with coordinated action expressing team cognizant action. As players navigate the match environment to achieve team goals, they perceive and act on shared affordances through coordinated movement calibration to ecological information. This 1-to-1 mapping between coordinated action and ecological information defines the players’ ability to realize shared affordances through coordinated effectivities [14–16].

Understanding team coordination is a challenge parallel to Bernstein’s problem of regulating degrees of freedom during movement [38]. However, instead of mechanically linked (e.g., limbs and muscles), players in a team are informationally linked (perceptively constrained) while coordinating their movements, maintaining a functional unit toward a goal—a *team synergy* [17]. Synergic self-organization among teammates does not require a central controller [39, 40]. Instead, team synergies are maintained by players' coordinated effectivities in relation to shared affordances, regulated by local perception–action couplings. Team synergies have measurable properties which express the embodied, embedded and goal-directed coupling between cognition and action in the team–environment system. In other words, team synergies express team cognizant action [17]. Importantly, team synergies and team cognizant action are not equivalent constructs. Team cognizant action refers to the ongoing perceptual-motor regulation of behavior at the team level, whereas team synergies represent the emergent patterns of coordination that result from this process. This indicates that team synergic properties provide  a way to operationalize the measurement of team cognizant action.

**Fig. 2:**
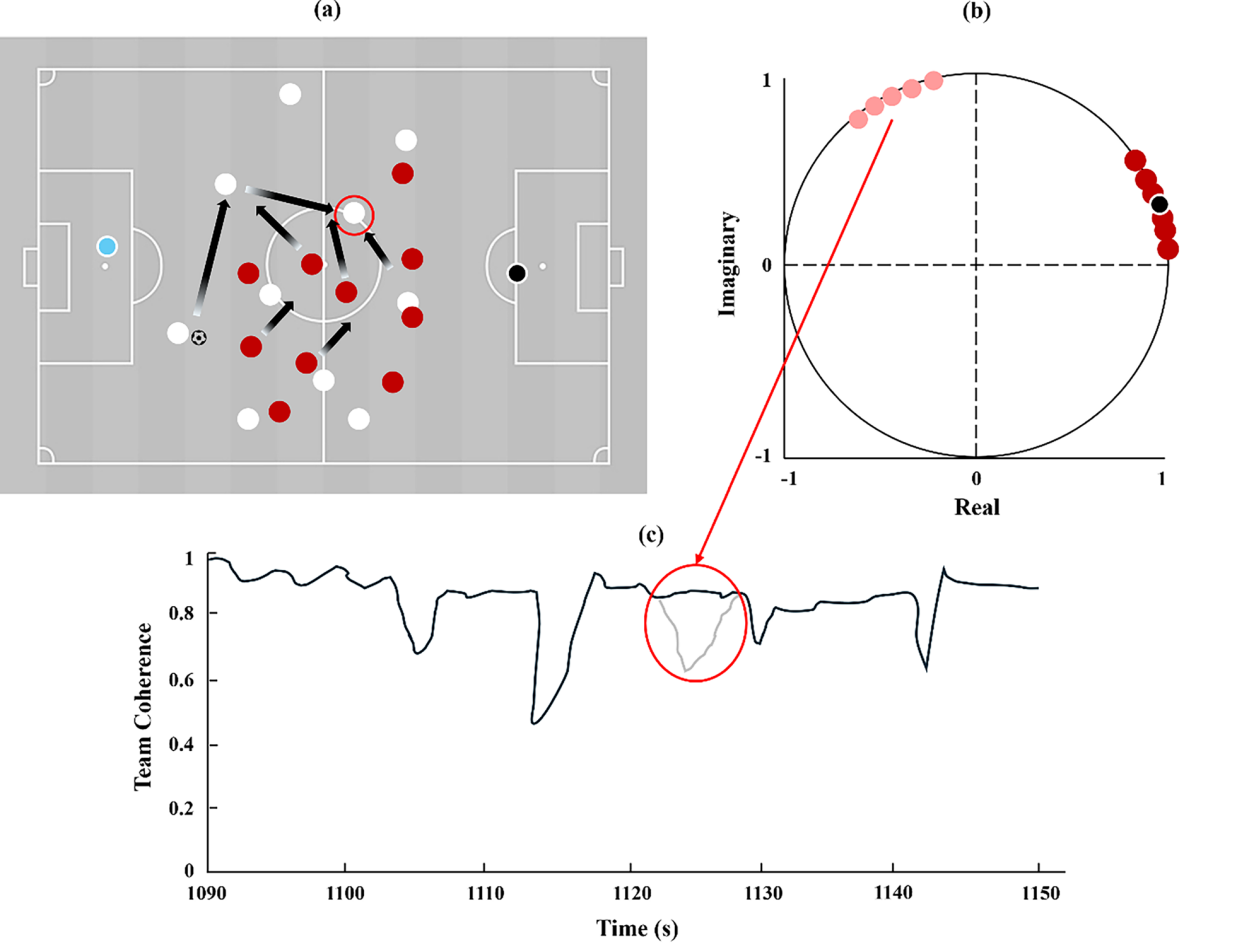
Perception–action couplings driving team synergies. **a** Defenders (red filled circles) maintain team coherence by adjusting their positions (black arrows) relative to the ball and the goal, forming a cluster (unit circle), as attackers (white filled circles) move the ball to find space to progress toward the goal. **b** The clustering phase of defender–ball–goal angles (unit circle at top-right) defines team coherence levels (bottom time series). **c** Coherence breaks (e.g., gray line between *t* = 1120 s and *t* = 1130 s, highlighted by the red large circle) represent moments when defenders fail to adjust their positions, resulting in some defenders being “thrown” from the cluster, as illustrated by pink circles in **b** (a dynamic version of this figure is provided in Online Resource 1)

Consider now a defensive scenario in which defenders coordinate their actions to prevent the opposition’s progression toward the goal, as illustrated in Fig. 2a.

To maintain team coherence (performance outcome), defenders form a team synergy through coordinated effectivities, adjusting their movements (e.g., accelerating and decelerating) to maintain functional interpersonal distances that constrain attacking space. This coordination is guided by shared affordances to block potential progression routes, which are specified by ecological information, such as relative positioning between defenders, the ball, and the goal.

Carrilho et al. [18] demonstrated, through cluster phase analysis (Fig. 2b) [18, 41, 42], how these coordinated effectivities acting on shared affordances preserved the team synergy, and how the team synergy broke down when defenders failed to adjust (Fig. 2c). Moreover, the authors were able to capture team synergic properties [17]: (i) reciprocal compensation, where defenders compensated for each other, (ii) interpersonal linkages, revealing role prominence and individual contribution to the team, (iii) degeneracy, defined as the ability of different elements to perform the same function [43], and (iv) dimensional compression, reducing multiple degrees of freedom to a collective variable [17, 18].

Notably, dimensional compression highlights fundamental differences across theoretical frameworks, helping to clarify how the ecological dynamics approach conceptualizes team cognizant action through team synergies. For instance, the active inference framework [44], grounded in the assumption of indirect perception, uses dimensional compression (or dimensionality reduction) to define system–environment boundaries (i.e., *Markov blankets* [45]), separating cognitive states internal to the system (e.g., players in a team) from external states (e.g., the match environment) [44, 46]. Within this approach, team behavior is analyzed in terms of how external information is processed to update internal models that guide collective action.

In contrast, within ecological dynamics, dimensional compression is not used to define boundaries but rather to reveal the organism–environment system as the relevant unit of analysis. This distinction reflects fundamentally different assumptions about the relationship between perception, cognition, and action, which have important implications for how team behavior is studied: either by modelling internal processes (e.g., active inference) or by quantifying the emergent coordination of the team–environment system through team synergies, which operationalize team cognizant action.

It is worth noting that the ecological scale of analysis, the organism–environment, can be examined across multiple, nested levels of organization (e.g., individual–environment, dyad–environment, subgroup–environment, team–environment) depending on the task and the ecological information that specifies goal-directed behavior. In the examples presented in this manuscript, such ecological information (e.g., stable relations in interpersonal distance, relative positioning, and collective spatial organization) specifies and constrains the team–environment system.

Operationally, the team–environment system is captured by eco-physical variables [47]—such as defender–ball–goal angles—which measure how players sustain functional relationships with task-relevant features of the match environment (e.g., ball, goal, opponents). If the team’s ability to block the opponent’s progression depends on maintaining a coherent positional relation with the ball and goal, then team performance is inseparable from the team’s coupling with the match environment. Eco-physical variables capture this coupling, avoiding the traditional organismic bias that isolates performance within the individual’s internal physiology, biomechanics, or psychology [12, 48]. In this sense, eco-physical variables provide a direct operational link between shared affordances, coordinated effectivities, and the emergence of team synergies.

Unlike constructs such as team mental models or collective intelligence, which are typically inferred from internal or aggregate properties, team cognizant action is directly observable in the continuous, coordinated interactions between players and their environment. Team synergicc properties, expressing team cognizant action within a team–environment system, provide a direct, continuous, and context-sensitive lens for understanding and improving team performance.

## Enhancing Team Cognizant Action Through Representative Design

To ensure that players develop behaviors in training that correspond to those in the match, theoretical guidance is needed. In this final section, we extend our theory into practice by outlining key heuristics for representative training design. Representative training design refers to the sampling of key informational variables from the performance environment to preserve perception–action coupling, so that behavior developed in training can correspond to competition [21, 50]. As previously discussed, coordinated effectivities refer to the disposition of different players to act upon shared affordances in pursuit of shared goals. This interdependency between goal-directed intentions [51], the perception of shared affordances, and the players' coordinated effectivities, forms the foundation for designing training environments that enhance team cognizant action. A robust theory of training design should, therefore, consider how these elements interact to shape team–environment couplings and guide effective coaching interventions [52]. Below, we present three heuristics informed by previous studies on behavioral correspondence and skill learning [22, 53]. These heuristics can be operationalized by three key design rules: (i) preserve information-movement couplings relevant to the target task, (ii) manipulate task constraints to stabilize affordance-effectivity adaptations, and (iii) evaluate performance through measures that capture team–environment coordination.

### Performance Achievement

Players' coordinated actions must be oriented toward achieving a shared task goal when simulating a specific match scenario, such as blocking the opponent’s progression. Rather than prescribing specific coordination patterns, practitioners should ensure that players maintain a shared intention throughout the exercise (education of intention [54]). This can be achieved by focusing on performance outcomes that assess whether players are effectively coordinating their actions to achieve the task goal. For example, instead of developing a training task focused on maintaining a predetermined spacing target between defenders, the focus should be on the performance outcome: whether defenders successfully block passing lanes and restrict ball movement within the defensive line, letting defenders adapt to each other to achieve the task goal, instead of providing rigid coordination instructions.

### Selecting Shared Affordances

Training tasks should allow players to perceive shared affordances that align with their common intention (i.e., achieve their shared goal). This means that the constraints of a training task create an ambient array of ecological information that is analogous to the environmental layout that is presented during the specific match situation being simulated.

For example, during a training task aimed at developing the behavior of blocking the opponent’s progression, defenders should regulate their positioning relative to key informational sources, such as attackers, the ball, and the goal [18]. These relations provide ecological information (e.g., relative distances and angles) that specifies shared affordances for blocking passing lanes and constraining space. As a result, defenders adjust their movements in a coordinated manner to maintain a functional defensive unit. If any of these key informational sources are missing, coordination is not possible, and the training task will not generalize to similar match situations. Thus, coaches should first identify the key sources of information that guide the players’ behavior in a particular situation (goals, ball, opponent, pitch location, etc.) and include these sources in their task design. Movement relative to these key information sources forms the ecological information to which players attune to perceive shared affordances.

For instance, consider a training task aimed at developing coordination within a back-four defensive line to create offside traps in a football match.^1^ Such a task should include key sources of information: (i) attackers attempting to break the defensive line, (ii) the defensive goal, and (iii) interchanging attackers in possession of the ball. In contrast, non-essential elements may be excluded (e.g., a second goal unless the task involves a transition to attack). However, if any of these key informational sources are missing, the ecological information available in the task will not correspond to that of the match. Specifically, attackers attempting to break the line and the location of the defensive goal specify the success of the defensive action (i.e., moving the line forward, in a coordinated manner, relative to the goal and the attackers), while the attacker in possession provides temporal information that specifies when the defensive line should move forward as a unit, typically in relation to the moment of the pass. Together, these sources of information constrain how defenders coordinate their movements to realize the shared affordance for an offside trap.

As players repeatedly engage with similarly structured ecological information, they become perceptually attuned to shared affordances representative of the match—i.e., they develop their sensitivity to specific information patterns related to configurations that involve the ball, opponents, and teammates. This is confirmed by previous research showing how futsal defenders become attuned to spatial–temporal information when trying to intercept passes. Attunement was captured as successful interceptions depended on defenders continuously regulating their movement velocity relative to the ball trajectory [55]. Moreover, research also showed how regular practice enhanced players' coordination in moving, as a cohesive unit, toward and away from the goal, when they perceived shared affordances soliciting them to coordinate their effectivities [56]. In every course of action, the team's skill is reflected in its players' shared perceptual attunement to the match environment, including their specific coordination with teammates. Ultimately, such shared attunement defines the team’s expertise [20, 54].

### Action Fidelity

Shared intentions and perceptual attunement guide players to precisely calibrate (i.e., scale movement to information) their coordinated effectivities to realize shared affordances. However, for this to occur, training tasks should promote action fidelity [22]. This step emphasizes that players’ actions should be coordinated during the training task in a manner similar to how they would be coordinated in the match. Returning to our task example, since ecological information in training is analogously structured to that in the competitive environment, defenders should perceive shared affordances to block and be able to apply appropriate forces and produce adjusted movement dynamics, such as speed and direction changes, to realize those affordances . If task constraints overly restrict movement, such as limiting space, defenders may not be able to move to effectively close down space as they would in a match.

Consider a training task aimed at developing lateral shifting of the defensive structure in response to a switch of play (i.e., a rapid lateral transfer of the ball to the opposite side). In this scenario, the change in ball location and relative positioning between defenders and attackers, relative to the goal, specifies a shared affordance for collective repositioning to keep blocking progression. To realize this affordance, defenders must coordinate their effectivities by adjusting their movements as a unit: the nearest defender accelerates toward the ball, while teammates synchronize their movements to maintain interpersonal distances and preserve a compact structure. This coordinated adjustment requires matching movement timing and magnitude across players.

To support this process in training, task constraints should ensure that defenders’ movements are representative of what is required of them in match situations. For example, if the available space is too small, the required acceleration magnitude will not correspond to match conditions, limiting players’ ability to coordinate their effectivities in a representative manner. Therefore, task design should preserve both the informational structure (e.g., ball movement, opponent positioning) and the movement demands (e.g., distance and speed of lateral shifts) that specify the shared affordance for coordinated defensive repositioning.

In summary, aligning task goals, shared affordances, and coordinated effectivities in training with those of the match context fosters shared perceptual attunement and collective movement calibration. Importantly, task constraints should be designed to preserve both the informational variables that specify shared affordances and the corresponding movement magnitudes required to realize them through coordinated effectivities. Manipulations of space, player numbers, or rules need to consider how players will experience representative information-movement couplings, thereby enhancing team cognizant action.

## Conclusions

This paper introduced team cognizant action as a proposed framework for understanding team behavior, emphasizing the inseparability of cognition and action within a team–environment system. Departing from psychological models that treat cognition as an inferential process and action as its execution, we proposed that team coordination emerges from interdependent perception–action couplings guided by shared affordances and coordinated effectivities. By measuring team synergistic properties through eco-physical variables, team behavior can be directly and continuously assessed during performance. This approach offers a parsimonious account of team behavior, with practical implications for training design. Specifically, it informs the application of the constraints-led approach [21] to team sports by emphasizing that task constraints can be designed to shape not only individual behavior but also team synergies that emerge from shared affordances and coordinated effectivities as expressions of team cognizant action.

## Supplementary Information

Additional file 1

## Data Availability

Not applicable.

## Footnotes

1 A back-four refers to a defensive unit composed of four players positioned horizontally across the pitch, typically forming the last line of defense in front of the goal. An offside trap is a coordinated defensive action in which defenders move forward as a unit to position attacking players in an offside position, thereby preventing them from receiving the ball legally.
